# Supplementary material for: Metabolomic Analysis Reveals Distinct Profiles in the Plasma and Urine Associated with IgE Reactions in Childhood Asthma
Source: J Clin Med. 2020 Mar 24;9(3):887. doi: 10.3390/jcm9030887 (PMC7141511; doi:10.3390/jcm9030887)
Supplement: Supplementary file 1 [file jcm-09-00887-s001.pdf]

Supplementary Materials

Supplementary Tables and Figures

Table 1. PLS-DA parameters and permutation test for distinguishing between children with asthma and controls, and between children with and without mite, food and IgE sensitization.

Atopic dermatitis	PLS-DA parameters				
	**Components***	**Q^2**	R^2	Q^2/R^2	$P_{permutation}$†
**Plasma**					
Asthma vs. Controls	1	-0.14	0.17	-0.84	0.472
Mite sensitization (with/without)	1	-0.16	0.21	-0.77	0.361
Food sensitization (with/without)	1	-0.06	0.12	-0.51	0.982
IgE sensitization (with/without)	1	0.04	0.24	0.17	0.578
**Urine**					
Asthma vs. Controls	1	-0.17	0.29	-0.59	0.455
Mite sensitization (with/without)	1	-0.19	0.27	-0.69	0.251
Food sensitization (with/without)	1	0.02	0.28	0.06	0.346
IgE sensitization (with/without)	1	-0.20	0.24	-0.82	0.775

PLS-DA, partial least squares-discriminant analysis; Q^2, predictive capability; R^2, correlation coefficients. *The number of components based on Q^2 indicates the best classifier of PLS-DA using a 10-fold cross-validation method. †1000 random permutations were performed.

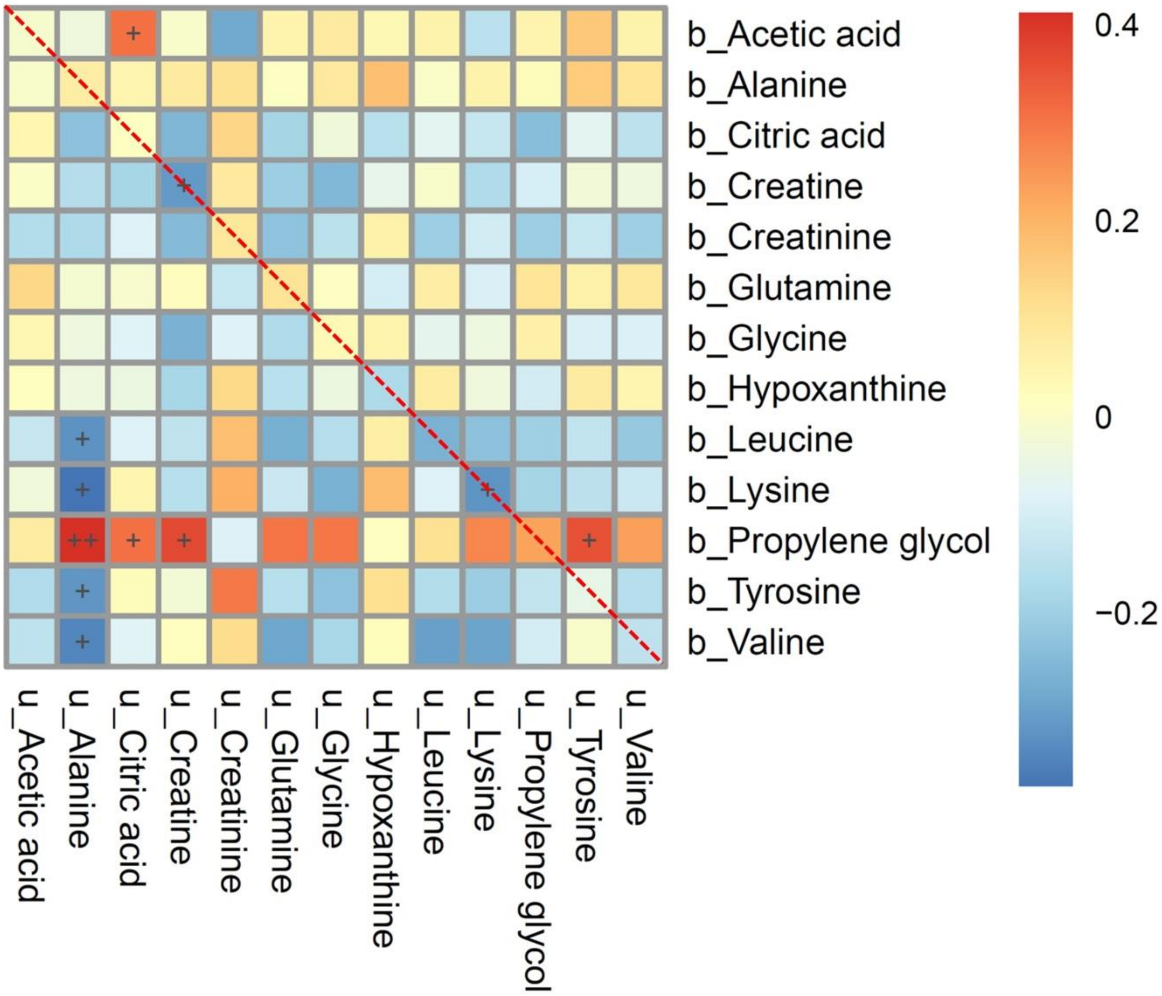

Figure 1. Heatmap of Spearman's rank correlations between metabolites both existed in plasma and urine. Color intensity represents the magnitude of correlation. Red color represents positive correlations; blue color represents negative correlations. + symbol means a *P*-value < 0.05; ++ symbol means a *P*-value < 0.01.

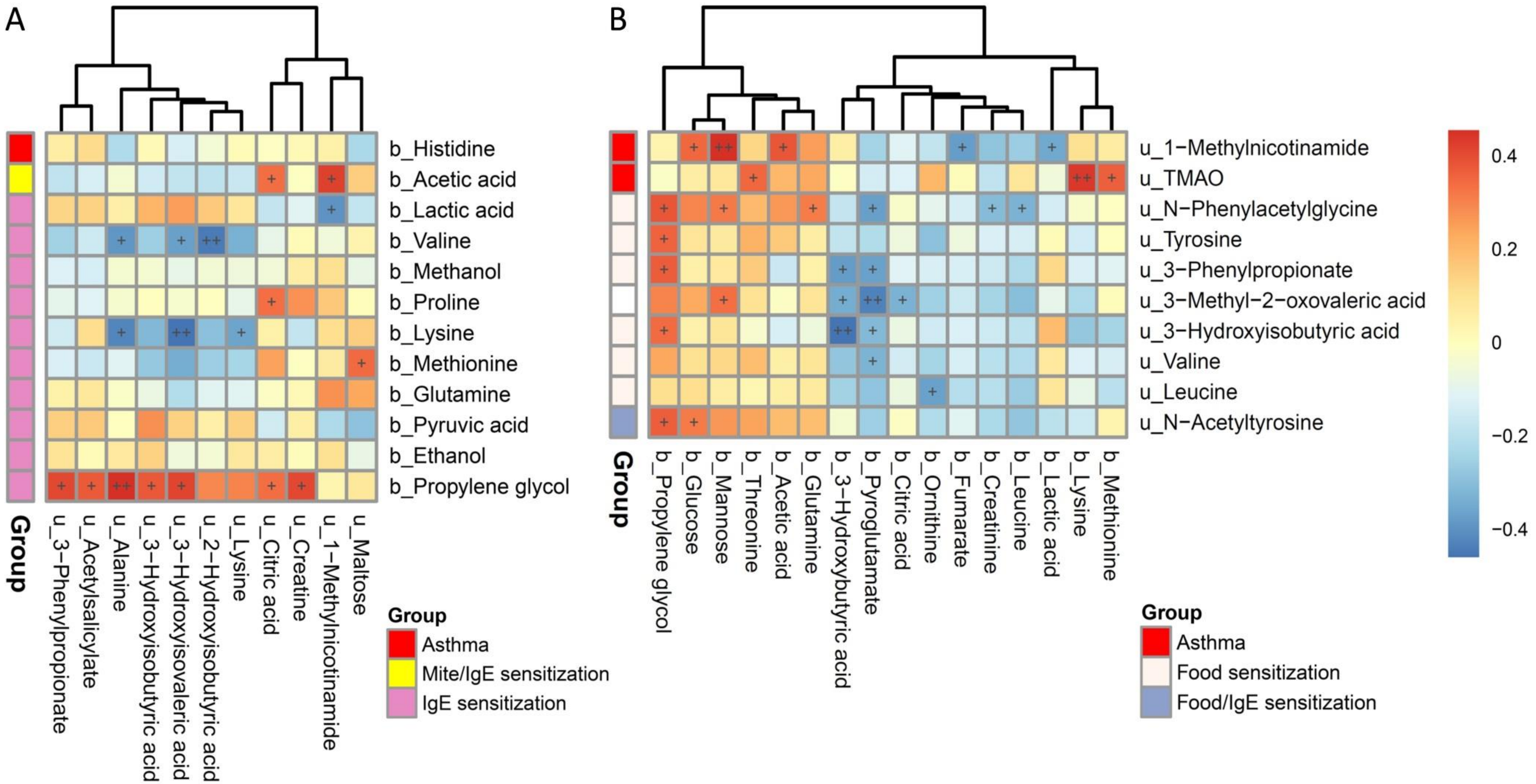

Figure 2. Heatmap of Spearman's rank correlations of atopic indices associated plasma and urine metabolites with urine metabolome (A) and plasma metobolome (B) respectively. Color intensity represents the magnitude of correlation. Red color = positive correlations; blue color = negative correlations. + symbol means a P-value < 0.05; ++ symbol means a P-value < 0.01.
